# Evidence of a bi-directional relationship between heart failure and diabetes: a strategy for the detection of glucose abnormalities and diabetes prevention in patients with heart failure

**DOI:** 10.1186/s12933-024-02436-3

**Published:** 2024-09-28

**Authors:** Paul Valensi

**Affiliations:** Polyclinique d’Aubervilliers, Aubervilliers and Paris Nord University, Bobigny, France

**Keywords:** Heart failure, Diabetes, Prediabetes, Insulin resistance, OGTT, HbA_1c_, Prevention, Recommendation, Sodium–glucose cotransporter 2 (SGLT2) inhibitors, Glucagon-like peptide-1 (GLP-1) receptor agonists

## Abstract

Prevalence of heart failure (HF) and diabetes are markedly increasing globally. In a population of HF patients, approximately 40% have diabetes which is associated with a more severe HF, poorer cardiovascular outcomes and higher hospitalization rates for HF than HF patients without diabetes. Similar trends were shown in HF patients with prediabetes. In addition, the association between HF and renal function decline was demonstrated in patients with or without diabetes. However, the exact prevalence of dysglycemia in HF patients requires further investigation aiming to clarify the most accurate test to detect dysglycemia in this population. The relationship between HF and diabetes is complex and probably bidirectional. In one way, patients with diabetes have a more than two-fold risk of developing incident HF with reduced or preserved ejection fraction than those without diabetes. In the other way, patients with HF, when compared with those without HF, show an increased risk for the onset of diabetes due to several mechanisms including insulin resistance (IR), which makes HF emerging as a precursor for diabetes development. This article provides epidemiological evidence of undetected dysglycemia (prediabetes or diabetes) in HF patients and reviews the pathophysiological mechanisms which favor the development of IR and the risks associated with these disorders in HF patients. This review also offers a discussion of various strategies for the prevention of diabetes in HF patients, based first on fasting plasma glucose and HbA_1c_ measurement and if normal on an oral glucose tolerance test as diagnostic tools for prediabetes and unknown diabetes that should be performed more extensively in those patients. It discusses the implementation of diabetes prevention measures and well-structured management programs for HF patients who are generally overweight or obese, as well as current pharmacotherapeutic options for prediabetes, including sodium–glucose cotransporter 2 inhibitors which are among the pillars of HF treatment and which recently showed a benefit in the reduction of incident diabetes in HF patients. Thus, there is an urgent need of routine screening for dysglycemia in all HF patients, which should contribute to reduce the incidence of diabetes and to treat earlier diabetes when already present.

## Introduction

Heart failure (HF) is the leading cause of morbidity and mortality in Western countries, with increasing prevalence worldwide. Diabetes is a well-recognized risk factor for HF, whether left ventricular ejection fraction (LVEF) is reduced or preserved. The recent Universal Definition and Classification of HF even categorized diabetes as among the most relevant risk factors for incident HF; other significant predictors for HF are hypertension, chronic kidney disease (CKD), and obesity [1]*.* As for HF, the global diabetes prevalence keeps increasing, with a current estimate of 9.3%, and is expected to rise further to 10.2% by 2030, as well as its financial burden [2, 3]. In 2021, there were 529 million people living with diabetes, with type 2 diabetes accounting for 90% of overall diabetes prevalence, and it is estimated that 50% of individuals with diabetes are currently undiagnosed [2, 4]. According to estimates, 10–30% of the population with diabetes have clinically manifest HF, while 30–40% of all cases of acute or chronic HF present with prevalent diabetes [5]. In the general population, HF was shown to be associated with a higher prevalence of diabetes compared to subjects without HF [5]. Among patients with HF, the presence of diabetes also aggravates the severity of HF and increases the risk of cardiovascular (CV) events, including CV mortality. Subjects with diabetes have a significantly higher risk for HF hospitalization (hHF) than subjects without diabetes [6, 7]. Interestingly, HF with preserved ejection fraction (HFpEF) is currently the most frequent form of HF. In diabetes, both HF and CKD frequently co-exist, aggravate each other and exert synergistic effects towards an increased risk of cardiac and renal events [1, 8, 9].

Even people with prediabetes are at higher risks of developing incident HF [10, 11] or incident CV disease [12]. Prediabetes also exposes to an increased risk of developing diabetes [13]. Regarding the definition of prediabetes there is some controversy, particularly regarding potential discrepancies between test thresholds as correct diagnostic tools for assessing Impaired Fasting Glucose (IFG), Impaired Glucose Tolerance (IGT), and hemoglobin A_1c_ (HbA_1c_), and between diabetes organizations [14, 15]. Dysglycemia is commonly seen in overweight and obese patients, although a large proportion of dysglycemia is unrecognized in those people due to failed screening tests if glycemia is measured only at fasting instead of doing an oral glucose tolerance test (OGTT) [16].

On the other way, the hypothesis that HF ​​itself may increase the risk for new-onset diabetes emerged nearly a decade ago [17]. As with diabetes, prediabetes and insulin resistance (IR) are also thought to develop more likely in patients with HF, even without other risk factors like overweight or hypertension compared with healthy individuals. In addition, both prediabetes and IR are associated with a more severe HF and an increased risk of all-cause mortality and adverse cardiac outcomes compared to patients with normal blood glucose levels [18–23]**.**

Thus, preventing diabetes in HF patients with prediabetes could appear as an attractive challenge [18]. Glucose abnormalities often remain undetected in HF subjects [24], highlighting the urgent need to perform more accurate diabetes screening with OGTT or at least fasting plasma glucose (FPG) and HbA_1c_ measurements in all HF patients. Therefore, the use of simple and reliable predictive tools and prevention programs should be encouraged to reduce the incidence of new diabetes in patients at risk [25]. Furthermore, the management of HF has significantly changed over the last decade, including the class of sodium–glucose cotransporter 2 inhibitors (SGLT2is) among the pillars of HF treatment [26]. This class might bear a metabolic interest in HF patients with prediabetes [27].

The aim of this review is to advance epidemiological and mechanistic understanding of the interlink between HF and diabetes, and to provide a guidance with measures that should be implemented to prevent the development of diabetes in subjects with HF.

## Epidemiological aspects and evidence of a deleterious reciprocal relationship between diabetes and HF

Diabetes and HF are closely interrelated: subjects with diabetes have an increased risk of developing HF across the entire range of glucose levels, and subjects with HF are at higher risk of developing diabetes.

### The presence of diabetes is associated with an increased risk of HF and worse outcomes among HF patients

Diabetes is highly prevalent among patients with HF, in both HFpEF and HF with reduced ejection fraction (HFrEF), yet the presence of HFpEF is more common in diabetes. About 42% of patients with HFrEF have diabetes, compared to about 45% of patients with HFpEF and this proportion has been increasing over the past 15 years in this population [28–30]. The incidence rate of HF in patients with diabetes has been rising, these patients having more than twice the risk of developing HF than nondiabetic individuals, this risk persisting after adjustment for age and relevant comorbidities [31–34]. Studies have also reported that diabetes independently increases the risk of HF up to twofold in men and fivefold in women compared with age-matched controls [7, 26, 35].

The association between diabetes and HF is seen even in people with recent-onset diabetes or younger age [36]. In patients with diabetes, advanced age, diabetes duration, obesity, presence of coronary artery disease (CAD), insulin use, CV risk factors such as hypertension and hypercholesterolemia are all independent risk factors for the development of HF. Among CV complications in people with diabetes, HF may be the first to manifest. Moreover, patients with diabetes but without evidence of clinical heart disease may display abnormalities of myocardial systolic contractility with diastolic dysfunction that have been demonstrated to correlate with increased HbA_1c_ levels [37]. In line with this finding, a study comprising 6688 adult subjects without prevalent CV disease who were followed for incident hospitalization for HF (HFpEF and HFrEF) during a median period of 14.9 years showed that HbA_1c_ and FPG in the diabetes range were associated with higher risks for HF of both types [38].

Patients with diabetes and HF with either preserved or reduced ejection fraction show increased mortality and morbidity rates compared with HF patients without diabetes. This increased risk is observed in those patients with diabetes and HF of either ischaemic or non-ischaemic origin [39]. Thus, diabetes increases the risk of HF and impairs the prognosis in HF patients.

Numerous clinical trials indeed showed that the presence of diabetes (priorly known or newly diagnosed) in patients with HF resulted in worse outcomes than in nondiabetic patients. Some of them (PARADIGM-HF (Prospective Comparison of ARNI With ACEI to Determine Impact on Global Mortality and Morbidity in Heart Failure) [21], CHARM (Candesartan in Heart failure– Assessment of Reduction in Mortality and Morbidity) [22], PARAGON-HF (Prospective comparison of ARni with Arb Global Outcomes in heart failure with preserved ejectioN fraction) [23]) demonstrated that diabetes is associated with worse clinical status and a significantly increased risk of adverse cardiovascular outcomes in patients with HF irrespective of ejection fraction phenotype compared with normoglycemic patients. Overall, the rates of the primary composite outcome of HF hospitalization and CV death were the highest in subjects with diabetes compared to those in other HbA_1c_ categories, i.e. normoglycemia and prediabetes. Some other CV outcome trials (CVOTs) which tested SGLT2is in HFrEF patients showed the same trend. Among the patients included in the Empagliflozin Outcome Trial in Patients with Chronic Heart Failure with Reduced Ejection Fraction (EMPEROR-Reduced) and the Dapagliflozin and Prevention of Adverse Outcomes in Heart Failure (DAPA-HF) trials, those with diabetes were also exposed to a higher risk of hHF and CV death compared to those without diabetes [40]. Along the same line, the exploratory analysis of the DAPA-HF trial [41] reported that among patients in the placebo group, the primary outcome, a composite of hHF and CV mortality, occurred in 25.5% of those with diabetes compared with 17.7% of those without diabetes. Also, according to the KaRen study [42], in patients with HFpEF, the prognosis was somehow as severe as in patients with HFrEF but the impact of comorbidities like diabetes was even greater.

Diabetic cardiomyopathy (DCM) is a condition that results from changes in the structure, function, and metabolism of the myocardium and cardiomyocytes in response to diabetes. All these changes that occur during exposure of the heart to chronic hyperglycemia and subsequent oxidative stress will contribute to cardiac dysfunction and increased risk for HF [43, 44]. The Heart Failure Association of the European Society of Cardiology (ESC) defines DCM as a left ventricular systolic and/or diastolic myocardial dysfunction in the presence of diabetes. Diabetes is rarely exclusively responsible for myocardial dysfunction, but usually acts in association with contributing factors such as obesity, arterial hypertension, CKD and/or CAD, causing additive myocardial impairment [45]. Several molecular mechanisms, numerous proteins and signaling pathways, and a myriad of cardiac metabolic abnormalities have been involved in the pathogenesis of DCM [46]. Recently, a novel molecular mechanism involving the JunD/PPAR-γ in cardiomyocytes has been shown to be associated with early lipotoxic diabetic heart dysfunction [47]. In patients with diabetes, the progressive pathogenic increase in JunD expression correlated with that of PPAR-γ and cardiac steatosis as evidenced by lipid accumulation (ceramide and triacylglycerol) measured in healthy hearts implanted in those patients [47]. Thus, DCM might account for the worse prognosis of HF in diabetic patients.

### Heart failure is thought to promote the progression to diabetes

The relationship between HF and diabetes also works the other way. People with HF are at increased risk for subsequently developing new-onset diabetes [48], in particular if they have a high body mass index (BMI) or waist circumference; smoke or have a history of smoking; have elevated glucose levels; inadequate blood pressure; take diuretic therapy, or have more symptomatic HF. The Cardiovascular Health Study (CHS) [17] examined the impact of HF at inclusion on incident diabetes after 3 or 4 years in a cohort of 3748 nondiabetic elderly subjects. Among the 3,165 subjects with normal fasting glucose at baseline, 80 had HF. Among the HF patients, 10% developed prediabetes *vs* 5.1% of patients without HF, and 6.2% developed diabetes *vs* 1.5% of patients without HF. Finally, 83.8% of patients with HF at baseline remained normoglycemic after follow-up compared to 93.4% of patients without HF. These data highlight the impact of HF status on the development of hyperglycemia within a few years. However, the CHS study had some limitations due to the limited number of patients with HF at baseline (80) out of more than 3,000 subjects and to a single fasting glucose measurement at baseline. Looking at the interventional studies in HF Table 1 summarizes the incidence rates of new diabetes. When focusing on the control arms, in the Studies of Left Ventricular Dysfunction (SOLVD), new-onset diabetes as defined by two measures of FPG ≥ 126 mg/dl developed in 22.4% of congestive HF patients from the placebo group during the mean follow-up of 2.9 years (35 months). However, this finding was reported in a still limited subset of 291 patients [49]. In the same line, in the SUPPORT (supplemental benefit of an angiotensin receptor blocker in hypertensive patients with stable heart failure using olmesartan) trial [50], the incidence rate of new-onset diabetes at approximately 1-year follow-up visit (as defined by plasma glucose ≥ 200 mg/dl at 2 h of an OGTT) was 5.5%, and much elevated if the patients with HF were prediabetic than if they were normoglycemic at baseline. Some recent trials provide interesting data in much larger populations of patients with HF. According to results of the DAPA-HF trial [51], in a large population of 2,605 patients with HF without known diabetes at baseline, over a median follow-up of 18 months, the incidence of new-onset diabetes (as defined by an HbA_1c_ ≥ 6.5% (48 mmol/mol) or clinical diagnosis) was 7.1% in the placebo group, and significantly higher if they had prediabetes than if they were normoglycemic at inclusion (95.5% of patients who developed diabetes had prediabetes at baseline based on the American Diabetes Association (ADA) criteria) [51]. The pooled analysis of the DAPA-CKD (Dapagliflozin and Prevention of Adverse Outcomes in Chronic Kidney Disease) and DAPA-HF trials included 4,003 patients with CKD or HF but with no history of diabetes and HbA_1c_ less than 6.5% at baseline. New-onset diabetes developed in 6.3% of patients from the placebo group during a median follow-up of 21.2 months [52]. Additional evidence for new-onset diabetes in individuals with CV disease comes from the DAPA-MI (Dapagliflozin in patients with MI) trial [53], which included 4,017 patients with acute myocardial infarction (MI), without chronic HF but with impaired left ventricular systolic function and without diabetes at baseline. A new diagnosis of diabetes occurred in 78 (3.9%) patients assigned to placebo during a median follow-up of 11.6 months. To note, the placebo-corrected mean change from baseline in body weight with dapagliflozin was − 1.65 kg (95% CI − 2.12 to − 1.18).

**Table 1:** Incidence of new-onset diabetes in HF interventional studies

Clinical trial	Participants with HF without diabetes at baseline n (%)	Mean or median follow-up Months	Criteria for new-onset diabetes	Incident diabetes n (%)	
SOLVD (N = 6,797) [49]	291 (4.3)	35 (mean)	Two measures of FPG ≥ 126 mg/dl	Placebo	Enalapril
				31/138 (22.4)	9/153 (5.9)
SUPPORT (N = 535) [50]	255 (47.7)	12 (median)	OGTT	Control arm	NA
				14/255 (5.5)	
DAPA-HF (N = 4,744) [51]	2,605 (55.0)	18 (median)	Two consecutive HbA_1c_* or clinical diagnosis	Placebo	Dapagliflozin
				93/1,307 (7.1)	64/1,298 (4.9)
Pooled analysis of DAPA-CKD and DAPA-HF (N = 4,003) [52]	4,003 (35.0% from DAPA-CKD and 65.0% from DAPA-HF)	21.2 (median)	Two consecutive HbA_1c_* or clinical diagnosis	Placebo	Dapagliflozin
				126/2,008 (6.3)	85/1,995 (4.3)
DAPA-MI (N = 4,017 patients with acute MI, without chronic HF but with impaired left ventricular systolic function) [53]	4,017 (100.0)	11.6 (median)	Investigator reported or clinical diagnosis	Placebo	Dapagliflozin
				78/1,998 (3.9)	42/2,019 (2.1)

*Based on the American Diabetes Association (ADA) criteria (≥ 6.5%, 48 mmol/mol) to define diabetes[15]

NA, not applicable

This is a lower rate compared to the incidence of new diabetes in patients with HF from the DAPA-HF and pooled DAPA trials. Thus, these data emphasize the risk of diabetes and the role of preexisting prediabetes in patients with HF.

Subjects at risk of diabetes are in particular those who already have moderate blood glucose abnormalities which are extremely common in HF patients. Patients with dysglycemia who develop new-onset diabetes are likely in a state of IR at baseline as this will be discussed below.

## Underdiagnosed dysglycemia and the risks associated in HF patients

Glycemic status is strongly associated with risk for incident HF, suggesting a continuous relationship between any blood sugar abnormality and HF risk and HF prognosis [54–56].

### Prevalence of unknown dysglycemia

In addition to the high prevalence of diabetes in patients with HF irrespective of ejection fraction phenotype, there is increasing evidence that both undiagnosed diabetes and nondiabetic dysglycemia (prediabetes) are also common in HF patients. Inadequate knowledge of glucose disturbances (or previously unknown abnormal glucose regulation), misdiagnosis of patients’ glycemic status, i.e. prediabetes (IFG, IGT) or undiagnosed diabetes that remains unidentified and misclassified as normoglycemia (NGT), could be explained by a lack of screening in a context of HF with frequent associated risk factors or use of diagnostic tests which are not the most reliable depending on the at-risk population (according to age, overweight, obesity, waist adiposity). The OGTT is recognized as the preferred reference diagnostic tool for prediabetes and unknown diabetes [16]. In contrast to HF, several studies reported the high prevalence of prediabetes detected by an OGTT in patients with other CV conditions, in particular CAD [57, 58] or after an acute coronary syndrome (ACS) [59], together with the publication of the consensus statement on the care of these patients which reported the OGTT as necessary for the appropriate classification of glucose tolerance in patients with ACS [60]. ESC guidelines further sustained the use of the OGTT in CV disease which is the only means of diagnosing IGT [26]. In HF, data are scarce (Table 2). In the ‘Randomized Evaluation of Strategies for Left Ventricular Dysfunction’ (RESOLVD) Pilot Study [20], there was a total of 663 HF patients included, of whom 176 (26.6%) had a diagnosis of diabetes at baseline. Of the remaining 487 nondiabetic patients, 111 (16.7% of the total) had elevated fasting glucose concentrations (≥ 6.1 mmol/l) while 53 (8%) of these patients had fasting glucose concentrations in the diabetic range (FPG ≥ 7·0 mmol/l). Overall, 287 (43.3%) patients (176 diabetic and 111 nondiabetic) had abnormalities of glucose metabolism by fasting blood glucose criteria. In the study population of Egstrup et al. [24], 309 patients with systolic HF with or without dysglycemia were followed during a median time of 591 days. At baseline an OGTT was performed in 227 of patients who were without a history of diabetes. Among these 227 tested individuals, 136 (44%) were classified as having NGT, 51 (16.5%) prediabetes (IGT), and 40 (12.9%) newly diagnosed diabetes. If an OGTT had not been performed and FPG ≥ 7.0 mmol/l had been used instead, all of the patients with IGT and 16 of the patients (40%) with newly diagnosed diabetes would have been misclassified as NGT. The patients with such glycemic disturbances defined by a prior diagnosis of diabetes or the results of an OGTT had older age and higher BMI compared with patients with NGT. Based on an OGTT performed twice, 113 (21.1%) patients with IGT and 3 (0.5%) patients with diabetes were newly detected in a cohort of 535 subjects with chronic HF in the control arm of a subanalysis study of the SUPPORT trial [50]. The study of Son et al. [18] proved that in a population of Vietnamese subjects with HF (HFrEF and HFpEF) but without diabetes, even free of overweight and hypertension, the prevalence of prediabetes as detected by an OGTT was significantly higher in HF patients compared to healthy controls of similar age, gender and body weight (63.2% *vs* 24.2%, respectively; *p* < 0.0001).

**Table 2:** High prevalence of diabetes and previously undiagnosed dysglycemia in HF patients

Clinical trial	HF phenotype	Total patients	Glycemic status assessment	Normoglycemia n (%)	Newly-detected prediabetes n (%)	Diabetes n (%)	
						Prior diagnosis	Newly-detected
RESOLVD (2000) [20]	HFrEF	663	FPG	376 (56.7)	58 (8.7) (IFG**)	176 (26.6)	53 (8)
Egstrup et al. (2011) [24]	HFrEF	309	OGTT in 227 patients	136 (44)	51 (16.5) (IGT)	82 (26.5)	40 (12.9)
SUPPORT (2019) [50]	~ 80% HFpEF	535	OGTT twice	142 (26.5)	113 (21.1) (IGT)	277 (51.8)	3 (0.5)
PARADIGM-HF (2016) [21]	HFrEF	8,274	HbA_1c_*	2,160 (26.0)	2,103 (25.5)	2,907 (35.0)	1,106 (13.5)
CHARM-preserved (2017) [22]	HFpEF	1,072	HbA_1c_*	189 (18.0)	217 (20.0)	428 (40.0)	238 (22.0)
CHARM-Alternative/Added (2017) [22]	HFrEF	1,578	HbA_1c_*	254 (16.1)	349 (22.1)	558 (35.4)	417 (26.4)
PARAGON-HF (2022) [23]	HFpEF	4,796	HbA_1c_*	1,534 (32.0)	874 (18.2)	2,062 (43.0)	326 (6.8)

*Based on the International Diabetes Expert Committee criteria: < 6%/6–6.4%/ > 6.4% or ≥ 6.5% (< 42/42–46/ > 46 or ≥ 48 mmol/mol) defining normoglycemia, prediabetes and diabetes, respectively

** IFG: 110–125 mg/dl (6.1–6.93 mmol/l)

Interesting data are available from intervention trials, mostly based on HbA_1c_ thresholds according to the International Diabetes Expert Committee criteria to define diabetes and prediabetes (Table 2).

Among the intervention studies, the PARADIGM-HF trial included 8,274 participants with chronic HFrEF. Of these, 2,907 (35%) had a history of diabetes, 1,106 (13.5%) had newly-detected diabetes, 2,103 (25.5%) had newly-detected prediabetes, and 2,160 (26%) had HbA_1c_ < 6.0% [21].

The CHARM program included 7,599 patients with both HFpEF and HFrEF [22]. Among the 2,650 tested individuals of the two trials, 986 (37.2%) had a history of diabetes, 655 (24.7%) had newly-detected diabetes, 566 (21.4%) were classified as newly-detected IGT, and 443 (16.7%) as NGT. The combined prevalence of newly-detected diabetes and prediabetes was high in both types of HF, but somewhat less in those with HFpEF compared with HFrEF (42% *vs* 48.5% of patients, respectively). The prevalence of prediabetic dysglycemia was more common than normoglycemia (HbA_1c_ < 6%) in both types of HF: 20% in subjects with HFpEF and 22.1% in those with HFrEF. The prevalence of newly-detected diabetes was also high, but was slightly less common in subjects with HFpEF compared with HFrEF (22% *vs* 26.4%). Conversely, the prevalence of known diabetes was slightly higher in patients with HFpEF (40% *vs* 35.4%). As a result, the prevalence of diabetes (known and newly-diagnosed) was 62% and 61.8%, among patients with HFpEF and HFrEF, respectively. Although similar prevalences of hyperglycemia in patients with HFpEF and HFrEF were found, differences between patients with the two phenotypes were found in respect of many of their baseline characteristics.

Among the 4,796 participants of the PARAGON-HF trial who had HFpEF (LVEF ≥ 45%), 49.8% had diabetes (HbA_1c_ ≥ 6.5%), among which those with newly-detected diabetes accounted for 6.8% of all participants. Moreover, 18.2% of all participants were diagnosed as IGT and 32% of those were tested as normoglycemic. Of note, 11% of participants with diabetes had a BMI < 25 kg/m^2^ ("lean diabetes", non-overweight individuals) [23]. The main findings were that in this population of HFpEF participants, diabetes affected around half of the patients and diabetes and prediabetes together accounted for 68% of the participants, highlighting the high prevalence of dysglycemia in this HF phenotype.

Altogether these studies confirm the remarkably high prevalence of dysglycemia, including previously undiagnosed diabetes and prediabetes, in patients with HF of both types. In the 4 above-mentioned trials this prevalence estimated based on HbA_1c_ measurement was 39% in means. Furthermore, overall the results show that FPG detects fewer of these patients than OGTT and HbA_1c_ (Table 2). However, the best comparison between these 3 tests would have been to carry them out within the same study. The importance of a need to better assess all HF patients for their glycemic status, including those without comorbidities, was underscored in a recent study [18].

### Risks associated with unknown dysglycemia

#### Priorly undiagnosed diabetes impairs the prognosis of HF patients

Some clinical trials provide insight into the risk of worsening HF in patients with diabetes, both known and priorly undiagnosed, which has been shown to be associated with poorer clinical status and a significantly increased risk of adverse CV outcomes (higher rates of hHF or death from CV causes). Among the glycemia categories, the adverse outcomes were the highest with diabetes compared with nondiabetic status and normoglycemia. Furthermore, previously known diabetes was evaluated at significantly higher risk for the primary composite outcome compared to both undiagnosed diabetes and prediabetes which were at higher risk compared with normoglycemia in CHARM [22]. Patients with diabetes (known and priorly undiagnosed) seemed to suffer from more severe HF in terms of higher New York Heart Association (NYHA) class and levels of N-terminal pro-brain natriuretic peptide (NT-pro-BNP) [24]. As regards mortality, known diabetes in systolic HF patients led to 29% of all deaths, which is a more than three-fold higher mortality rate than patients with normoglycemia (9%), while priorly undiagnosed diabetes resulted in an intermediate mortality rate of 15% [24].

#### Prediabetes impairs the prognosis of HF patients as well

In nondiabetic individuals with HF, elevated fasting glucose was shown to be associated with a more severe HF accompanied by more severe cardiac symptoms [20]. Even before the diagnosis of diabetes, prediabetes has been shown to be associated with adverse CV outcomes in HF patients at an intermediate risk between that observed in diabetes and normoglycemia [21–23]. The results of the SUPPORT trial [50] also indicated that prediabetes was associated with poor prognosis in terms of hHF, MI, and all-cause death when complicated by albuminuria in HF patients. According to the exploratory analysis of the DAPA-HF trial [41], among the patients with HFrEF without diabetes at baseline in the placebo group, 68% had prediabetes (HbA_1c_ 5.7–6.4%) and 32% had a normal HbA_1c_ (< 5.7%). The primary outcome (the composite of an episode of worsening HF or CV death) occurred in 18% of patients with prediabetes and 16.9% among those with normoglycemia. In DAPA-HF [51], the risk of death from any cause in patients who developed new-onset diabetes during follow-up was more than twofold that of patients who did not progress to diabetes. The event rates were 16.6 and 7.2 per 100 patient-years, respectively, with an adjusted HR of 1.70 (95% CI 1.04–2.80; *P* = 0.035) remaining significant after adjustment for baseline variables and treatment assignment. Similar trends were observed for death from CV causes. Considering the total number of hHF and CV deaths, the event rates were 28.6 and 14.6 per 100 patient-years in those with and without new-onset diabetes, respectively (unadjusted HR 1.90, 95% CI 1.18–3.05; *P* = 0.008). However, after adjustment, this was no longer significant (HR 1.37, 95% CI 0.83–2.24; *P* = 0.22). When considering CV death only as a separate outcome, the event rates were 14.7 and 5.8 per 100 patient-years in patients who developed new-onset diabetes and those who did not progress to diabetes. After adjustment, this heightened risk remained significant (adjusted HR 1.77, 95% CI 1.04–3.02; *P* = 0.035). Furthermore, in the pooled analysis of the DAPA-CKD and DAPA-HF trials [52], in the placebo arm, the group of patients with prediabetes experienced a greater number of events per patient-years related to the primary composite compared with the normoglycemic status (6.2 *vs* 0.6). Prediabetes was further reported to be a marker of worse cardiac function in patients with HF of both types. HF patients with prediabetes were more often in high NYHA class and had higher NT-pro-BNP level, a lower LVEF than those with normoglycemia [18]. Prediabetes exposed to intermediate mortality compared with the other glycemic status [24].

#### Prediabetes is associated with a very high risk of incident diabetes; is this risk greater in HF population than in the general population?

In addition to CV outcomes, prediabetes is critical as a precursor of diabetes. People with HF who have prediabetes have a very high risk for new-onset diabetes (Table 3). In the placebo arm of SOLVD, based on FPG measured on average 7.9 times during follow-up, the incidence rate of diabetes was 48% among the patients with prediabetes and 17.3% in those with normoglycemia at baseline. In the control arm of SUPPORT, based on the OGTT performed one year later, the incidence rate of diabetes was 11.5% in the patients with prediabetes and 0.7% in those with normoglycemia at baseline. As previously mentioned, the pooled analysis of DAPA-CKD and DAPA-HF trials [52] reported that in the placebo arm, 6.3% of the patients with HF or CKD developed new-onset diabetes, and more than 90% of them had prediabetes at inclusion based on the ADA criteria; the incidence rate of diabetes was 9.7% in the patients with prediabetes and only 1% in those with normoglycemia at baseline. In the same line, the randomized controlled trial with empagliflozin in HF with a reduced EF [61] found that 80 (12.6%) patients in the placebo group with prediabetes at baseline developed a new-onset diabetes during a median follow-up of 16 months (Table 4). The trial with empagliflozin in HF with a preserved EF [62] further showed that 137 (14%) patients with prediabetes at baseline in the placebo group developed a new diabetes after a median follow-up period of 26.2 months. A little more than 10% of subjects with prediabetes in means in the placebo groups of these 3 trials testing SGLT2is developed diabetes during the period of follow-up which is consistent with an estimate of 10.6% of the world adult population with IGT [63] (Table 4).

The risk of developing dysglycemia in HF population seems to be higher than in the general population. The SUPPORT trial which was carried out in Japan found that among HF subjects, the incidence rate of newly-diagnosed IGT and diabetes was 16.7/1,000 person-years and 11.2/1,000 person-years, respectively [50]. This incidence rate of newly-diagnosed diabetes was higher than in the general population (8.8/1,000 person-years). In 2000, the annual rates of progression to diabetes from IGT in the general population ranged from 2.3% per year to ~ 11% per year with higher rates in non-white racial/ethnic groups [64]. An estimate of around 5–10% per year of people with IGT progressing to diabetes was later reported [65, 66]. A large sample size and representative study of adult workers from Spain [66] showed that 23% of 23,293 subjects who had prediabetes at baseline progressed to diabetes after 5 years of follow-up. This corresponded to a 4.6% mean annual rate of progression to diabetes. Prediabetes was diagnosed based on one parameter (FPG) and measured HbA_1c_ when the FPG was elevated. No OGTT was carried out. In CHS, among the patients with prediabetes at baseline, HF increased the risk of developing diabetes in multivariate analyses (Odds ratio (OR) 2.44 [95%CI 1.44–4.16]). Specifically, a history of HF was associated with developing prediabetes or overt diabetes at follow-up in multivariate analyses [17]. As previously underlined, studies defining glycemic status according to OGTT are missing in HF population.

Thus, the incidence of new-onset diabetes in patients with HF is far higher in patients with prediabetes than in normoglycemic patients. Together, these results highlight the greater attention that should be paid to prediabetes in individuals with HF to prevent the risk of progression to diabetes.

#### Early detection of diabetes is important to prevent the other complications of diabetes

Once established, diabetes can lead to several complications. Microangiopathic complications suffered by diabetic persons include retinopathy, nephropathy, neuropathies, a variety of cardiovascular problems including HF, and skin ulceration [67]. Major achievements have been made over the past three decades in reducing the risk of those complications through optimal control of glycemia, blood pressure, and lipids. In fact, avoiding the transition to diabetes appears to be the best way to prevent the various complications. Several trials have already shown the effectiveness of lifestyle interventions (healthy diet, weight loss, and increased physical activity) in reducing the risk of conversion from IGT to incident diabetes [13]. Other treatment options for prediabetes include pharmacotherapy (use of weight loss inducing drugs or glucose-lowering drugs) and bariatric surgery [13, 67]. Thus, people with prediabetes (known or suspected due to an association with risk factors such as obesity, hypertension, older age, larger waist circumference) are at higher risk of diabetes and should benefit from better monitoring and detection as a result of simple and reliable screening tests [16]. This will be further detailed in the review.

HF exposes to more renal failure [68] and patients with HF often present with renal dysfunction [1, 42, 69], especially in the population with diabetes [21, 23, 40, 41, 70]. The association between HF and CKD aggravates each other and exerts synergistic effects towards an increased risk of major cardiac and renal events [1]. In the DAPA-CKD study which included patients with albuminuria and low estimated glomerular filtration rate (eGFR) [71], 13% and 8% of patients with and without diabetes, respectively, had a history of HF. Furthermore, after a median follow-up of 2.4 years, the incidence rate of hHF or CV death was three-times higher in the patients with diabetes. An elevated risk of decline in renal function (eGFR decrease of 50% or more, end-stage renal disease, or death due to renal failure) has been observed in association with diabetes (known and undiagnosed) and even prediabetes in several CVOTs [1]. In an exploratory analysis of DAPA-HF study [51], after a median 18-months follow-up period, patients with incident diabetes had more severe decline in kidney function characterized by lower eGFR, and higher BMI compared to patients with HF who remained nondiabetic. Looking at the placebo arm of this trial [41], a kidney adverse event was reported in 8.7% of patients with diabetes and 6% among patients without diabetes. Also, in EMPEROR-Reduced trial, the renal prognosis was more severe in patients with than without diabetes, with two-fold higher rates of decline in eGFR and renal outcomes, and major risk for renal failure (chronic dialysis, renal transplant or sustained reduction of ≥ 40% eGFR) [70]. Among the glycemic statuses, diabetes was associated with the highest risk for the composite renal outcome compared to prediabetes for whom the risk was moderate and compared to normoglycemia which had the lowest rate, as shown in PARADIGM-HF [21] and PARAGON-HF [23], and the elevated risk was apparent across the spectrum of EF albeit nonsignificantly so in patients with EF > 35% and tended to be accentuated at lower EF in patients with HFrEF [21].

These observations highlight the importance of preventing kidney decline in HF patients and early detection of diabetes to reduce associated complications. This issue will be detailed later in the review.

#### Newly-detected dysglycemia should be part of the stratification of HF-related events

Prediabetes is linked with numerous chronic complications [72] including HF which was previously shown to be more severe in prediabetic patients with HF of both types compared with normoglycemic patients. Therefore, early screening is of paramount importance to assess glycemic status in all HF patients without previously known diabetes in a more routine way in order to reduce the risk of incident diabetes [18]. The choice of test plays a major role in diagnosing the patient's glycemic status, depending on whether it is diabetic, prediabetic or normoglycemic as the result may contribute to treatment decision and patient outcome. Prediabetes is currently defined by IFG (100 − 125 mg/dl, 5.55–6.93 mmol/l) and/or IGT (2 h plasma glucose (PG) during a 75 g OGTT = 140 − 199 mg/dl, 7.77–11.04 mmol/l) and/or a HbA_1c_ level between 5.7 and 6.4% (39–46 mmol/mol), according to the ADA classification [15]. Some epidemiological studies demonstrated that in patient populations [16, 24], simplifying screening by measuring only FPG or HbA_1c_ offers low sensitivity and fails to identify the same subjects as does the OGTT. Therefore, a large proportion of prediabetic statuses (IGT) and undiagnosed diabetes could remain unidentified and misclassified as NGT. There is evidence that the OGTT is considered to be the best test for identifying early stages of hyperglycemia and asymptomatic type 2 diabetes. In [18], based on OGTT results, the prevalence of prediabetes was significantly higher in HF patients compared to control subjects. In addition, there is substantial evidence supporting the 1-h PG level ≥ 155 mg/dl (8.6 mmol/l) for earlier detection of prediabetes. Robust scientific and clinical data indicate that 1-h PG appears to be more relevant and a better predictor for identifying high-risk individuals than FPG, 2-h PG or HbA_1c_, and this test is highly recommended in screening patients with CV disease for referral to diabetes prevention programs [72].

## Insulin resistance in HF patients

IR, with or without diabetes, has been suggested to promote an elevated risk for HF. Conversely, as with the relationship between HF and incident diabetes, patients with HF have a highly significant risk for IR.

### High prevalence of IR in HF patients, independent from overweight

Insulin resistance is a highly prevalent metabolic disorder in HF patients, independently of an ischemic etiology, whether ischemic or non-ischemic [73]. Fasting hyperinsulinemia and IR in patients with both HF etiologies were reported many years ago [73, 74]. Many such patients have been thought to have IR prior to developing left ventricular systolic dysfunction. Several lines of evidence have suggested that HF is an IR state [75]. IR is common among nondiabetic patients with HF and may be found in 33–70% of them [76]. More severe IR has been observed in patients with HFrEF than in those with HFpEF, as shown in a study which assessed IR within the physiological range of insulin–glucose interaction by using the short insulin sensitivity test (SIST) in 40 patients with HFrEF or HFpEF and 20 controls [77]. Despite lack of certainty regarding the precise relationship between IR and HF, epidemiological findings suggested that IR may predict the subsequent development of HF, independent of all established risk factors, including diabetes [78]. Further supporting evidence also suggested that IR precedes HF rather than being a consequence of it, as proinsulin levels (a surrogate marker of IR) were reported to be higher in patients who subsequently developed HF than in control patients 20 years before their HF was diagnosed [79].

In the ARIC (Atherosclerosis Risk in Communities) study [80], a community cohort of 12,606 subjects without diabetes, prevalent HF, or history of MI at inclusion was analyzed to assess the relationship between IR and incident HF. IR was associated with an increased incident HF, however the risk began earlier than the traditionally used HOMA-IR (Homeostatic Model Assessment of Insulin Resistance) index cut-point of 2.5. In a series of 663 stable HF patients including more than 40% with known diabetes or hyperglycemia, those with IR and with or without diabetes were shown to have more advanced symptoms of HF and a shorter 6-min walk distance compared to nondiabetic patients without IR [20]. When examining according to functional status, the nondiabetic patients in NYHA class III/IV had higher glucose and insulin levels than patients in NYHA class I/II.

Patients with HF are more insulin resistant compared to patients with CAD and normal controls. The study of Swan et al. [73] assessed insulin sensitivity in patients with chronic HF and its relation to disease severity. A group of 38 patients with a wide range of disease severity and different etiologies and a second control group of 21 patients with CAD were included. A high degree of IR was observed in patients with chronic heart failure with a 58% reduced mean insulin sensitivity and a 131% increase in fasting insulin levels compared with data in the healthy control group of 20 patients. This study demonstrated that HF is associated with marked IR and is characterized by both fasting and stimulated hyperinsulinemia. The cause of IR in HF is unclear and was not directly addressed by this study. Whether IR is a cause, or a consequence of HF remains uncertain, but the idea that IR progresses within the natural course of HF was suggested by this study.

Obesity, a primary determinant of IR, has also been independently associated with incident HF [80]. However, the study of Son et al. [18] reported that even without overweight, the prevalence of IR in patients with HF was higher compared to controls without HF. The study included 190 patients of Vietnamese origin with both HFrEF or HFpEF and 95 healthy controls. The prevalence of IR, assessed using several indexes, was doubled in HF patients compared to controls. The prevalence of IR as defined according to HOMA-IR was significantly higher in patients with HF than in the control group and was also higher in patients with HFrEF than with HFpEF (58.8% and 50.0%, respectively *vs* 26.3%). The population characteristics of this study were that the patients were nondiabetic, not overweight, without impairment of renal function. Dysglycemia was also more prevalent in patients with IR, but the patients were free of hypertension and lipid disorders, the other components of the metabolic syndrome and had similar BMI and waist circumference as the control subjects. As IR was highly prevalent despite normal body weight and no excess in abdominal adiposity and was not associated with other metabolic disorders, IR was more likely the consequence rather than the cause of HF and this might then lead to dysglycemia. The high incidence of new diabetes in HF patients is consistent with this hypothesis. However, the glycemic alterations induced by IR, ranging from mild dysglycemia to diabetes, may conversely increase the risk of HF and HF progression as summarized above.

### IR in HF according to the ischemic or non-ischemic cause

Patients are clinically classified as having HF of ischemic or non-ischemic etiology based on a history of MI or on objective evidence of CAD such as angiography or functional testing. According to the ESC guidelines, establishing HF etiology should constitute the primary step, essential for planning an appropriate therapy [81].

Ischemic HF is determined by the presence of one or more obstructive plaques, which result in a reduced coronary blood flow, causing myocardial ischemia and subsequent impairment of myocardial contraction and relaxation. Attention paid to the role of microcirculation in the pathophysiology of ischemic heart disease and HF is growing. Dysfunction of coronary microvascularization determines an inability of coronary circulation to satisfy myocardial metabolic demands, due to the imbalance of coronary blood flow regulatory mechanisms, including ion channels [82]. These alterations lead to the development of hypoxia, fibrosis and tissue death, consequently determining a loss of myocardial function, even beyond the presence of atherosclerotic epicardial plaques [83]. In contrast, microvascular angina is a condition characterized by restricted blood flow to the heart without involvement of obstructive plaques in the coronary arteries. Microvascular angina was found to be associated with IR, glucose intolerance, and hyperinsulinemia [84, 85].

A very high rate of non-ischemic HF patients without known pre-existing diabetes is seen with IR compared with a matched healthy control population [78, 86, 87]. Patients with non-ischemic HF are even more insulin-resistant than patients with CAD [73]. In addition, patients with HF caused by non-ischemic heart disease have a high prevalence of glucose dysmetabolism. For instance, patients with idiopathic dilated cardiomyopathy are characterized by both elevated hyperinsulinemic and hyperglycemic responses after oral glucose load [87].

The association between the degree of IR and both severity and etiology of chronic HF was further investigated in a study in which 38 patients with a wide range of clinical severity of chronic HF were examined. In the study of Swan et al. [73], the etiology of HF was ischemic heart disease in 21 patients and idiopathic dilated cardiomyopathy in 17 other patients. Those with chronic HF due to CAD were more likely to have abnormalities in glucose metabolism than were patients with chronic HF due to idiopathic dilated cardiomyopathy. Patients with ischemic heart disease and normal left ventricular function were found to be insulin resistant and hyperinsulinemic but to a significantly lesser degree than patients with chronic HF due to ischemic heart disease. To note, a number of baseline characteristics were found to differ significantly between the ischemic and non-ischemic HF cohorts, with ischemic patients more likely to be older, male, Caucasian, smokers, and to have more comorbidities such as hypertension and diabetes [88, 89].

### IR is associated with worsening HF

IR with nondiabetic hyperglycemia may be associated with increased symptoms of HF, and a greater risk of CV events, including worsening HF and hHF [73, 90]. IR was reported to be an independent predictor of mortality among nondiabetic patients with HF. In a prospective study of 105 male subjects with HF, lower insulin sensitivity assessed by an intravenous glucose tolerance test using the minimal model technique was shown to predict higher 2-year mortality in HF patients and this independently of body composition and established risk factors [91]. This suggests that impaired insulin sensitivity may play a role in the pathophysiology of HF progression. Recently, a post-hoc analysis of the UKPDS (UK Prospective Diabetes Study) also found that in patients with newly-diagnosed diabetes a doubling of homeostatic model assessment-2 for IR (HOMA2-IR) was associated with a 5% greater risk of HF/death outcomes [56]. Interestingly, there was a trend for a more pronounced difference in HF severity according to the presence or absence of IR than according to the presence or absence of prediabetes. Therefore, IR should also be considered for risk stratification in HF patients [18].

Alike diabetes, IR worsens HF prognosis, independent of other variables, including peak oxygen consumption (VO_2max_) and LVEF, implying that IR is pathogenic rather than simply a marker for worsened HF. IR is tightly associated with more severe HF and adverse outcomes. Some studies showed that IR increased significantly with worsening NYHA class and was significantly associated with a lower exercise capacity and VO_2max_ as well as endothelial dysfunction; thus, increased IR was significantly related to increased severity of HF and IR seems to progress with increasing severity of HF [20, 73, 92]. In our population of Vietnamese patients, those with IR had a more severe HF, being more often in high NYHA class with higher NT-proBNP level and lower LVEF than those without IR [18]. Existing evidence shows that IR is associated with LV remodeling which might contribute to adverse outcomes in patients with HF [80, 93–95]. IR also plays a role in chronic HF by inducing myocardial changes. In particular, heart vulnerability to ischemia and pressure load are increased in response to the use by the myocardium of more free fatty acids (FFAs) and less glucose due to cardiac metabolism changes. The presence of IR in patients with HFrEF who are not diabetic also compromises recovery of ejection fraction [96]. Also, increased IR in patients with HF may lead to impaired peripheral vasodilatation and increased left ventricular afterload, causing further impairment of cardiac performance [90]. In addition, hyperinsulinemia leads to activation of sympathetic nervous system (SNS) and subsequently elevates the levels of adrenergic neurotransmitters (norepinephrine, epinephrine and dopamine) which have vasoconstriction properties [97], in turn worsens IR and cardiac energy metabolism.

Also, IR may impair the myocardium structure. Myocardial changes that result from chronic hyperinsulinemic state in IR are cardiac hypertrophy, collagen formation and fibrosis of the myocardium [76, 98]. Formation of advanced glycosylation end products occurring in the myocardium may also lead to increased collagen cross-linking and myocardial stiffness [48].

**Table 3:** Incidence of new-onset diabetes in patients with HF according to glycemic status

	Glycemic status assessment	Mean or median follow-up Months	Number of measures n	New-onset diabetes in patients with normoglycemia* at baseline n (%)	New-onset diabetes in patients with prediabetes* at baseline n (%)
SOLVD, placebo arm (N = 138) [49]	FPG	35 (mean)	7.9	19 (17.3)	12 (48.0)
SUPPORT, control arm (N = 255) [50]	OGTT	12 (median)	1	1 (0.7)	13 (11.5)
Pooled analysis of DAPA-CKD and DAPA-HF, placebo arm (N = 2,008) [52]	HbA_1c_ Two consecutive HbA_1c_ values ≥ 6.5% or clinical diagnosis	21.2 (median)	2	8 (1.0)	118 (9.7)

*HbA_1c_ criterion: < 5.7% (39 mmol/mol) and 5.7–6.4% (39–46 mmol/mol) for normoglycemia and prediabetes, respectively

**Table 4:** Effects of SGLT2 inhibitors on the risk of a new-onset diabetes in patients with prediabetes

Clinical trial	Drug	Median follow-up Months	Sample size n	Incident diabetes n (%)		Events per 100 patient-years n		Hazard ratio (95% CI)
				Placebo	SGLT2 inhibitor	Placebo	SGLT2 inhibitor	
EMPEROR- Reduced (2020) (N = 3,730) [61]	Empagliflozin	16	1,268	80 (12.6)	71 (11.2)	10.6	9.3	0.86 (0.62–1.19)
EMPEROR-Preserved (2021) (N = 5,988) [62]	Empagliflozin	26.2	1,979	137 (14)	120 (12)	7.4	6.1	0.84 (0.65–1.07)
DAPA-CKD and DAPA-HF (2022) (N = 4,003) [52]	Dapagliflozin	21.2	2,408	126 (6.3)	85 (4.3)	6.2	4.2	0.69 (0.52–0.91)

### Mechanisms involved in IR among HF patients

The exact mechanisms of IR in chronic HF are not known. Similar to the interconnection between diabetes and HF, the relationship between IR and HF might work both ways. Then, HF may contribute to IR. Several possible explanations on the overlapping pathophysiological processes in both conditions have been suggested for the association of IR with HF as a pair of intricate disease [26].

SNS and renin–angiotensin–aldosterone system (RAAS) are activated in a compensatory manner by HF. This activation leads to increased FFAs released from adipose tissue and increased plasma glucose associated with a pancreatic damage mediated by cytokines (such as tumor necrosis factor alpha (TNF-alpha) and interleukins). The deleterious metabolic consequences of hyperglycemia and hyperlipidemia (glucotoxicity and lipotoxicity in the β-cells) may result in susceptible persons in reduced β-cell capacity for insulin secretion (“β-cell exhaustion”) which is associated with a diminution in β-cell mass through apoptosis. In the transition from IR to diabetes, 20% to 40% of islet cell mass is lost through apoptosis [48]. In addition, β-cell mass may be replaced by the accumulation of amyloid fibrils, which may lead to endoplasmic reticulum stress when insulin is overproduced resulting in nitric oxide (NO)-induced apoptosis [48]. Elevated catecholamines levels in chronic HF also contribute to aggravate IR.

Lower physical activity and a sedentary lifestyle which affect most HF patients have long shown to be associated with IR involving a cascade of molecular mechanisms. In response to physical inactivity, endurance-trained runners experienced a reduction in insulin action which occurred quickly through lowering levels of glucose transporter type 4 (GLUT4) expression in skeletal muscles [99]. The study by AlZadjali et al. [92] showed that in response to exercise test, the nondiabetic HF patients with IR had a significantly lower exercise duration, peak VO_2_, and peak cardiac output compared with patients without IR. A regular physical activity has demonstrated in general population great benefits in reducing the risk of IR and improving whole-body insulin sensitivity. Muscle contraction during exercise stimulates improvements in insulin sensitivity whose molecular mechanisms are linked to the translocation of GLUT4 to the cell membrane and thereby increasing glucose uptake [100].

## How to prevent diabetes in HF patients?

### Determine the glycemic status in all HF patients

Based on the above observations, avoiding the progression from prediabetes to diabetes in HF patients seems crucial and feasible. This implies first to use the appropriate screening tests. The detection of dysglycemia should be performed more extensively in patients with HF, based on the OGTT or more simply on FPG and HbA_1c_ measurements, in particular in those with other risk factors such as family history of diabetes, overweight, obesity, excess in waist circumference, age, which may be integrated in scores [13, 101]. Screening for prediabetes and diabetes by only FPG or by the OGTT as a diagnostic tool has been debated. In an overweight population, the OGTT has been shown to better detect a large proportion of prediabetes (70%) and undiagnosed diabetes (71%) that would remain unidentified if only FPG was used in selected populations [16]. Numerous studies support the OGTT as the reference diagnostic tool for the diagnosis of dysglycemia, but in HF population studies, detection of glucose disturbances was mostly based on HbA1c criteria. As previously described, glycemic status in HF patients may be assessed considering different criteria. In HF populations, the OGTT and HbA1c as well seem able to detect more patients with dysglycemia than FPG alone (Table 2). To note, the OGTT is the unique means to characterize IGT.

Together, these findings highlight the importance of the potential value of screening all patients with HF in order to detect glycemic abnormalities. This simple intervention could permit identification of patients with HF at high-risk of developing diabetes, who should benefit from intervention strategies aiming to reduce the risk of incident diabetes.

### The detection of prediabetes should lead to implement measures to prevent diabetes in HF patients

#### Lifestyle intervention

Some studies demonstrated that HF patients with prediabetes at baseline had, among manifestations of worse HF status, a lower mean KCCQ-CSS (Kansas City Cardiomyopathy Questionnaire—Clinical Summary Score) compared to patients with normal HbA_1c_ (higher scores indicating fewer symptoms and physical limitations associated with HF) [21, 23]. They also had higher BMI and more obesity and older age.

Through lifestyle intervention strategies, it is possible to reduce the risk of conversion from prediabetes to diabetes. Trials of lifestyle intervention included weight reduction, a healthy diet, increased physical activity, psychological support, coaching, questionnaires of follow-up. The rate of undiagnosed IFG/IGT is very high since only 4.8% out of 34.6% of study subjects (n = 1,547) with prediabetes who were a representative sample of the U.S. population received a formal diagnosis from their physicians [102]. This rate of diagnosis was assessed 3 years after publication of the Diabetes Prevention Program (DPP) (the National Health and Nutrition Examination Survey [NHANES] IV). Such people with IGT were included in lifestyle intervention trials (the DPP in the U.S. [102] or the Finnish Diabetes Prevention Study (DPS) [103, 104]) dedicated to the prevention or delay of progression to diabetes. The nonpharmacologic lifestyle intervention program used in the DPS had well-demonstrated efficacy and showed a risk reduction by 58% of conversion from IGT to diabetes [105]. People who are identified by primary care physicians as being overweight or obese should be referred to intensive diet and physical activity behavioral counseling programs for managed intentional weight loss. This observation is supported by the 3-year results of the intensive lifestyle intervention used in the DPS that showed significantly greater weight reduction in the intervention group as compared with the control group, -3.5 kg *vs* -0.9 kg, respectively [103]. Long-term follow-up of the randomized DPS further demonstrated improved lifestyle and decreased diabetes risk over 13 years [104].

In the same line, the China DQDPS (Da Qing Diabetes Prevention Study), the longest lifestyle intervention trial (median: 6 [DQDPS] *vs* 3.5 [DPP] and 4 [DPS] years), was designed to determine whether diet and exercise interventions in individuals with prediabetes may delay the development of type 2 diabetes, i.e., reduce the incidence of type 2 diabetes, and thereby reduce the overall incidence of diabetic complications, such as CV, renal, and retinal disease, and the excess mortality associated with these complications. The study, started in 1986, included 577 individuals with prediabetes who were randomly assigned to one of three interventions (diet, exercise, or both) or to serve as a control [106]. Over a 6-year period, the different intervention groups had incidence rates of diabetes 25–50% below that of the control group. In the Da Qing Diabetes Prevention Outcome Study which followed up participants from the original DQDPS, the reduction in diabetes incidence was seen to be remained for several years after the period of active intervention. Later on, the outcomes from 540 (94%) participants with prediabetes were assessed after 30 years of follow-up [107]. A median delay in diabetes onset of 3.96 years was seen in the intervention group. Significant reductions of 26% in CV disease events, 35% in microvascular complications, 33% in CV mortality, and 26% in all-cause mortality were reported, leading to an increase of 4.82 years in median survival and a mean increase of 1.44 years in life expectancy in the intervention group compared with control. Furthermore, a post-hoc analysis of the DQDPS reported the relationship between long-term beneficial health outcomes and duration of maintaining non-diabetes status after a diagnosis of prediabetes which may be influenced by the duration of lifestyle intervention, after a prediabetes diagnosis [108]. This analysis showed that individuals after a long-term follow-up period of 30 years, who remained nondiabetic for at least 4 years after a diagnosis of prediabetes, had a significantly lower risk of all-cause mortality, CV disease events, and microvascular complications compared to those who progressed to diabetes. A lower risk for CV mortality became significant at the end of 6 years. However, this preventive effect was not observed in participants who remained nondiabetic for a shorter period. The beneficial influence of lifestyle interventions on the DQDPS outcomes was in contrast with DPP in which adverse CV events were not reduced during the 21-year follow-up, despite long-term prevention of diabetes [109]. One possible explanation is that the different durations of the interventions across the two diabetes prevention studies may have affected the time of remaining without diabetes, which is associated with the long-term risk of death and vascular complications. Therefore, a longer diabetes-free time is associated with a better CV prognosis and might lower the risk of long-term adverse outcomes [108]. Long-term follow-up of the original DPP study might clarify these findings [109]. Of note, the Da Qing cohort was a higher-risk population with a greater proportion of smokers, a higher prevalence of hypertension, and diabetes with more severe hyperglycemia and a higher overall CV disease event rate.

Regarding the potential reversal from prediabetes to normoglycemia, in a study including 2,005 individuals with a diagnosis of prediabetes at baseline who were randomized to the lifestyle intervention group or the control group, the short-term effect of lifestyle intervention in those people with prediabetes was evaluated [110]. After a follow-up of around 1 year, there were 36.7% participants in the intervention group and 34.5% participants in the control group with normoglycemia. The decrease in FPG levels and waist circumference was significantly higher in the intervention group than in the control group. Participants who were younger or female with lower BMI and PG levels at baseline were more likely to reverse to normal glucose levels.

The role of a 5–7% weight loss was considered as determinant for an effective prevention. Reduction in weight has indeed been demonstrated as a key treatment strategy in overweight or obese population. PLIS (Prediabetes Lifestyle Intervention Study) evaluated weight loss-induced remission of prediabetes in 1,160 participants among general population. Subjects were randomized to either a control arm (based on standard lifestyle support similar as in DPP study) or an intensified lifestyle intervention for 12 months. Responders were defined as people who reversed to normoglycemia. In the lifestyle intervention group, 43% were responders as compared to 19% only in the control arm. All participants who were included in this analysis lost at least 5% of their body weight at baseline. Responders were characterized by an improvement in insulin sensitivity and reduced visceral adipose tissue compared to non-responders. Finally, responders had a 73% lower risk of developing diabetes than non-responders during 2 years after the study ended. Together, these findings showed that lifestyle interventions may help maintain diabetes-free time in people with prediabetes and that mechanisms of weight loss-induced return to normoglycemia in people with prediabetes in turn prevented the development of diabetes. Therefore, remission of prediabetes should be the primary therapeutic aim in individuals with prediabetes [111]. However, no such study specifically exists in HF patients, and the benefit of diabetes prevention by lifestyle intervention on the reduction of CV outcomes in patients with or without HF needs to be evaluated prospectively.

#### Weight loss-based prevention, but with caution in HF

The development of glucagon-like peptide-1 receptor agonists (GLP-1 RAs) has revolutionized obesity therapy management due to their ability to achieve marked weight reduction (more than 15% in means) and improve clinical outcomes. In SCALE (Satiety and Clinical Adiposity-Liraglutide Evidence), a 56-week study that involved 3,731 obese and overweight patients, 3.0 mg/day of subcutaneous liraglutide was associated with a great reduction in body weight up to more than 15% of their body weight in means, improved metabolic control and health-related quality of life. A total of 63.2% of the patients in the liraglutide group lost at least 5% of their body weight compared with 27.1% in the control group, and 33.1% and 10.6%, respectively, lost more than 10% of their body weight. A reduction of evolution to diabetes and regression from prediabetes to NGT was also observed [112]. The STEP-HFpEF (Semaglutide Treatment Effect in People with Obesity and Heart Failure with Preserved Ejection Fraction) trial randomized 529 obese patients with HFpEF and no known diabetes to injectable semaglutide 2.4 mg weekly (n = 263) *vs* placebo (n = 266). The median BMI was 37.0 kg/m^2^ at baseline. Treatment with semaglutide produced large improvements in HF-related symptoms, physical limitation and exercise function, and reduced inflammation and body weight across BMI categories, and the magnitude of benefit was directly related to the extent of weight loss [113]. Targeting obesity in HFpEF is of upmost interest, given the large evidence suggesting a potential causal role for adiposity in the genesis of HFpEF. Next, a similar benefit with semaglutide in diabetic patients with HFpEF was demonstrated in the STEP-HFpEF DM (Semaglutide Treatment Effect in People with Obesity and Heart Failure with Preserved Ejection Fraction and Diabetes Mellitus) trial [114]. Furthermore, the pooled analysis of STEP-HFpEF and STEP-HFpEF DM included 1,145 patients. Semaglutide was associated with greater benefit as compared with placebo in reducing body weight, and improving HF-related symptoms and physical limitations in participants with obesity-related HF with preserved ejection fraction [115]. The SELECT (Semaglutide Effects on Cardiovascular Outcomes in People with Overweight or Obesity) trial investigated the effects of injectable semaglutide 2.4 mg/week in patients (n = 17,604) with preexisting CV disease and overweight or obesity but without diabetes. The results showed an improvement in prognosis, with semaglutide being superior to placebo within a mean follow-up of 39.8 months in reducing the incidence of death from CV causes, nonfatal MI, or nonfatal stroke, and at the same time inducing a mean weight loss of 9.39% with semaglutide and 0.88% with placebo [116]. Semaglutide and liraglutide at high-dosage (3 mg/day) are approved by both the Food and Drug Administration (FDA) and the European Medicines Agencies (EMA) as pharmacologic treatments for obesity or can be prescribed to overweight subjects with comorbidities [117–119]. However, data regarding the potential of GLP-1 RAs in diabetes prevention specifically in HF patients are not available. Other GLP-based therapies are becoming available and deserve being tested for weight loss and diabetes prevention in HF patients.

Bariatric surgery is a highly successful method to achieve substantial weight loss in obese people. Moreover, in the HF population specifically, the relationship between obesity and HF makes the concept of using bariatric surgery to improve cardiac function, enable for left ventricular assist device or transplantation candidacy in those HF patients who struggle with qualifying for these advanced therapies based on BMI [120]. Bariatric surgery has also shown an association with a lower risk of adverse outcomes in obese patients with HF (all-cause mortality, hHF, and atrial fibrillation) [121].

The weight effect in patients with diabetes on SGLT2i was investigated in the DECLARE-TIMI 58 (Dapagliflozin Effect on Cardiovascular Events–Thrombolysis in Myocardial Infarction 58) trial that included 17,134 patients stratified into five BMI categories. The placebo-corrected mean weight loss with dapagliflozin was − 1.90 kg (average of 5 mean absolute differences between treatment groups). To note, the absolute changes in weight between treatment groups were slightly greater in higher BMI groups. Across the range of BMI, the SGLT2i dapagliflozin demonstrated larger CV and renal benefits among obese patients in reducing obesity-related outcomes including hHF and atrial fibrillation or flutter [122].

However, while obesity is clearly a risk factor for HF, a higher BMI has a paradoxical association with a decreased risk of mortality in patients with HF [123–125]. For example, in the CHARM program, underweight or low BMI (3 categories from 29.9 to < 22.5) was associated with increased mortality, primarily in patients without edema. The association between BMI and mortality was not altered by left ventricular ejection fraction, age, or smoking status [123]. With respect to weight loss and physical activity, the OPTICARE XL CR program showed promising results among patients with heart disease and obesity. This cardiac rehabilitation (CR) program may initiate a new pathway to secondary prevention of CV disease [126]. CR centers might offer such opportunities to HF patients of starting a diabetes prevention program. It’s time to raise awareness of physicians and healthcare professionals about recommendations and well-structured management programs to assist patients with HF and obesity to lose weight and, thereby, improve multiple risk factors and long-term prognosis [127].

#### Glucose-lowering drugs-based prevention

Metformin

Metformin is the reference drug to reduce insulin resistance and may be used in HF. According to the REACH (Reduction of Atherothrombosis for Continued Health) study, administration of metformin to patients with diabetes and established atherothrombosis was associated with a lower mortality rate compared to patients who did not receive metformin, 6.3% *vs* 9.8%, respectively [128]. Among subgroups of patients having shown this benefit were those with a history of congestive HF. In addition, metformin improves outcomes in patients with HF [76, 95]. This drug was shown to reduce the progression from IGT to diabetes in the DPP [102, 129]: 7.8 cases per 100 person-years of new-onset diabetes as compared with 11.0 in the control group, corresponding to a reduction in diabetes incidence by 31%, as compared with control [129]. However, metformin has not been specifically tested for diabetes prevention in HF patients.

Glitazones

Glitazones are efficient in diabetes prevention but contraindicated in HF due to their effect on increasing volemia. A study showed that pioglitazone was associated with a reduction in risk conversion of IGT to diabetes by 72% with significantly reduced levels of fasting glucose, 2-h post-OGTT glucose, and HbA_1c_ as compared with placebo. However, this drug was also associated with significant weight gain and edema [130]. In this study, a total of 602 patients were randomly assigned to receive pioglitazone (n = 303) or placebo (n = 299). After a median follow-up period of 2.4 years, the incidence of diabetes was 5.0% in the pioglitazone group and 16.7% in the placebo group. Conversion to normal glucose tolerance occurred in 48% of the patients in the pioglitazone group and 28% of those in the placebo group [130]. A washout period of 11.4 months further determined whether it was a true prevention effect or simply a treatment effect. After discontinuation of therapy, the protective effect of pioglitazone on incidence of diabetes attenuated and as a result, 23.0% of patients in the pioglitazone group remained as NGT *vs* 13.8% of those in the placebo group. The rate of IGT conversion to diabetes 11.4 months after cessation of therapy was similar in both treated and placebo groups. However, the cumulative incidence of diabetes from time of initial randomization to end of follow-up at 11.4 months remained lower in the treated group *vs* placebo group.

Sodium–glucose cotransporter 2 inhibitors

SGLT2is are more recent glucose-lowering oral drugs originally approved for use in patients with diabetes requiring additional glycemic control beyond metformin. SGLT2is also provide concomitant benefits for cardiovascular and kidney outcomes, regardless of the presence or absence of diabetes, as well as reduced blood pressure, then could have a great global health benefit [51, 63]. Several mechanisms are involved to account for this benefit [131]. Regarding the benefit of SGLT2is in the prevention of HF and in the improved prognosis of patients with HF, SGLT2 itself might play a role. Indeed, the expression of SGLT2 mRNA and protein in cardiomyocytes from end-stage failing heart and its overexpression in diabetic cardiomyocytes suggest that SGLT2 may be implicated in the pathogenesis of HF including cardiac steatosis. By preventing this overexpression, SGLT2is may have a direct and protective impact on metabolic pathways within cardiomyocytes, which might constitute one additional favorable mechanism involved in the improved prognosis of HF patients [132].

In subjects with diabetes, SGLT2is improve glucose control through various mechanisms including an improvement of insulin sensitivity and secretion, improvement hepatic insulin sensitivity, and enhancement of pancreatic β-cell function through protection of cells from glucose toxicity. As described above this class seems able to reduce the conversion from prediabetes to diabetes in HF patients, and then to reduce the incidence of new-onset diabetes. Indeed, there is evidence that SGLT2is reduce the risk of a new-onset diabetes in patients with prediabetes and HF or CKD (Table 4). Future trials among HF patients without diabetes should clarify the predictors of incident diabetes among those with prediabetes and which patient phenotype is the most likely to benefit of SGLT2 inhibitors for diabetes prevention. However, the question of a class effect remains at present unclear. Actually, the EMPEROR-Preserved and EMPEROR-Reduced trials did not demonstrate a statistically significant benefit of empagliflozin for diabetes prevention among patients with HF and prediabetes, although the hazard ratios were consistent with benefit [63]. Also, a higher incidence of new-onset diabetes in the placebo arm of EMPEROR studies (-Reduced and -Preserved) than of DAPA-HF (12.6% and 14% *vs* 7.1%) was noticed, but differences in patients’ characteristics at baseline and predictive factors of benefit should be carefully examined. In DAPA-HF, there was no heterogeneity in the effect of dapagliflozin based on most key prespecified subgroups, except for age and baseline NT-proBNP levels. Analyzing and comparing trials with different SGLT2is at the level of individual patient data could shed light on a potential heterogeneity in efficacy among this class of drugs.

Interestingly, DAPA-HF trial showed a reduction in incident diabetes but without a significant effect on mean HbA_1c_ levels during the trial between the active therapy and placebo groups, in both participants with prediabetes and participants with normoglycemia [51]. In addition to these mechanisms leading to improved glycemic control, some others including the depression of sympathetic activity [133, 134] and improvement of physical activity (suggested by higher scores on the KCCQ) are likely to contribute to the reduction of IR and might be involved. However, studies with SGLT2is dapagliflozin and empagliflozin also have some limitations, including the absence of glucose and insulin measures and of any assessments after a drug washout period. The fact that HbA1c, the biomarker of average glycemia, did not change argues for a real prevention; however, the patients were not followed after the end of the study to demonstrate whether the effect persisted. Nonetheless, the findings of DAPA-HF may provide insights into the underlying effect of SGLT2 inhibition on β-cell dysfunction in the transition from prediabetes to diabetes, and this class of drugs clearly represents an attractive therapeutic option for diabetes prevention. Further prospective studies are needed (1) to address whether the diabetes prevention benefit is a class effect and (2) to test the effects of SGLT2is on long-term clinical outcomes.

Glucagon-like peptide-1 receptor agonists (GLP-1 RAs)

Glucagon-like peptide-1 receptor agonists are a class of drugs used to treat type 2 diabetes and also at higher dosages to induce weight loss in obese patients as above mentioned. GLP-1 RAs reduce body weight, glycemia, blood pressure, postprandial lipemia and inflammation in affected subjects, thus actions that could contribute to the reduction of CV events. Several CVOTs have demonstrated that GLP-1 RAs reduce the rates of major adverse CV events in patients with diabetes. Mechanistically, the expression of GLP-1 receptor at low levels in the heart and vasculature raises the possibility that GLP-1 might have both direct and indirect actions on the CV system [135]. Some examples of drugs of this class include semaglutide, liraglutide, and dulaglutide. According to the ADA, metformin remains the preferred first-line therapy for treating diabetes. However, the addition of a GLP-1 analog should be considered in some conditions, such as in people with a contraindication or intolerance to metformin, or in subjects who do not reach their HbA_1c_ target in 3 months, and importantly in those with atherosclerostic cardiovascular disease independently of their effect on glycemic control [136, 137]. This class of medications helps improve IR, which might contribute to alleviate HF-associated outcomes [76, 95]. However, GLP-1 RAs were not tested at anti-hyperglycemic dosage for diabetes prevention.

Dipeptidyl peptidase-4 inhibitors (DPP-4is)

DDP-4is improve blood glucose regulation by increasing the active levels of incretins, GLP-1 and glucose-dependent insulinotropic peptide (GIP). By preventing the deactivation of GLP-1 and GIP, they increase insulin release and decrease glucagon levels. These drugs may reduce β-cell apoptosis and preserve β-cell function, and might thus prevent the progression from prediabetes to diabetes. Among this class, saxagliptin has showed an improvement in glycemic status of obese patients with IGT after 12 weeks of treatment and turned it to NGT status in the majority of patients [138]. An improvement of glucose variability was also reported after 8 weeks of treatment by sitagliptin or vildagliptin in patients with type 2 diabetes [139]. Furthermore, 12 weeks of sitagliptin treatment was recently shown to improve glucose tolerance and lipid profile in overweight individuals with prediabetes [140]. DPP-4is may then be suggested for diabetes prevention in the management of prediabetes. This class of drugs has been reported to be neutral with regard to the incidence of HF except for saxagliptin which was associated with an increased risk for hHF in the SAVOR-TIMI 53 (Saxagliptin and Cardiovascular Outcomes in Patients with Type 2 Diabetes Mellitus) randomized trial, mostly in patients with HF history or elevated plasma NT-pro-BNP levels without clear explanation for that [141].

Insulin

Despite the potential effect of basal insulin glargine on normalizing FPG levels, there was concern about whether insulin use might be linked to incident HF. The ORIGIN trial (Outcome Reduction With Initial Glargine Intervention) allocated 12537 people with IFG, IGT, or diabetes to receive insulin glargine or standard care [142, 143]. Assessment reported the effects of allocation to glargine on death, the composite of hHF or death, and recurrent episodes of hHF. Therapy with basal insulin glargine during a median follow-up of 6.2 years had a neutral effect on CV outcomes, including both initial and recurrent hHF, and cancers, and no increased CV risk in the patients with a history of HF. This intervention reduced new-onset diabetes: approximately 3 months after therapy was stopped, new diabetes was diagnosed among 30% in the insulin-glargine group *vs* 35% in the standard-care group of 1,456 participants without baseline diabetes. However, among the adverse effects, basal insulin glargine increased the risk of hypoglycemia, although the absolute increase in risk was low, and the rates of severe hypoglycemia were 1.00 *vs* 0.31 per 100 person-years in the standard-care group. Insulin glargine also modestly increased weight, by 1.6 kg in means whereas weight fell by 0.5 kg in the control group. Overall, these findings were clearly reassuring for people using or considering the use of insulin but this approach does not seem attractive for the prevention of diabetes.

When aiming diabetes prevention, patients could be more compliant to drug therapy even if this option is less effective than lifestyle interventions, adherence to lifestyle changes being often poor despite extensive support [13]. Ultimately pharmacological therapy combined with diet and exercise counseling may be a realistic option for reducing diabetes incidence [144].

#### Other drugs

RAAS blockers

RAAS inhibitors include angiotensin-converting enzyme inhibitors (ACE inhibitors), angiotensin-receptor blockers (ARBs), and direct renin inhibitors. ACE inhibitors and ARBs commonly have beneficial effects on patients with CV risk factors (hypertension, diabetes), with heart diseases (HF, CAD, MI) and CKD. These medications exert favorable effects on metabolism regulation. Interestingly, the results of six large-scale clinical studies have reported a remarkably consistent 14–34% reduction in the incidence of diabetes in hypertensive patients treated with ACE inhibitors for 3–6 years [145]. In line with this encouraging finding, the large, prospective, placebo-controlled randomized clinical trial whose primary outcome was the prevention of diabetes, DREAM (Diabetes REduction Assessment with ramipril and rosiglitazone Medications) was initiated. This trial investigated the effects of the ACE inhibitor ramipril 15 mg/day (n = 2,623) *vs* placebo (n = 2,646) on the development of diabetes or death (primary outcome) and on the regression to normoglycemia (secondary outcome) in adults aged 30 years or more with IFG and/or IGT, and no previous CV disease. After a mean follow-up of 3 years, the use of ramipril did not significantly reduce the incidence of diabetes or death but significantly increased regression to normoglycemia. Thus, the routine inhibition of the RAAS may not be indicated in individuals not at high risk for CV disease for the express purpose of preventing diabetes [146].

Statins and the risk of new diabetes

Some meta-analyses have showed that statin treatment is associated with a small increase, approximately 9–12%, in the risk for incident diabetes [147, 148]. However, evidence also suggests that its benefit in CV prevention outweighs this detrimental effect, especially in subjects with higher CV risk. Relevant guidelines should provide practical guidance for primary care physicians on the use of statins in subjects with or at risk of having new-onset diabetes [149] such as those with HF.

#### Information of the patient and his/her health care professionals

Awareness is requisite in terms of knowledge of prediabetic stage which represents an early and promising target to reduce the transition to diabetes or revert to normal blood glucose levels. For this, it is time to increase the awareness among medical doctors (MDs), cardiologists and diabetologists, in particular regarding the more systematic screening of HF patients, and their management by a multidisciplinary care team [150].

Because diabetes and CV disease are adverse conditions among individuals at higher metabolic risk for developing these disorders, clinical practice guidelines have been developed according to European [13, 26] or American [15] evidence-based recommendations. These guidelines aim to provide recommendations on risk factors, interventions promoting lifestyle changes, screening tools, diagnosis and detection, and diabetes prevention. They are intended to assist health professionals in proposing the best management strategies for each patient, and to advise organizations and funders on these issues. Testing is of particular importance for people with prediabetes. Comparison between HbA_1c_ and OGTT among patients addressed in cardiac rehabilitation units after an ACS demonstrated HbA_1c_ overdiagnosed prediabetes (52% *vs* 30%) and underdiagnosed normoglycemia (39% *vs* 58%). Additionally, HbA_1c_ was shown to have low sensitivity and specificity for the detection of prediabetes (IFG and/or IGT) at 64% and 53%, respectively. Thus, OGTT remains necessary for the correct diagnosis of blood glucose abnormalities in coronary patients [59] and this test, alone or associated with HbA_1c_, should be extended more routinely to patients with HF as well.

SGLT2is are clearly now one of the pillars for HF treatment [137]. Empagliflozin, dapagliflozin, or canagliflozin are recommended in patients with diabetes and CV disease, or at high CV risk, to reduce CV events. Empagliflozin is recommended in patients with diabetes and CV disease to reduce the risk of death [26]. SGLT2is are now indicated for use in patients with HF and diabetes [137]. The preventive effect of these drugs on diabetes in the population with HF deserves being considered in addition to their CV benefit.

## Conclusions

**Fig. 1:**
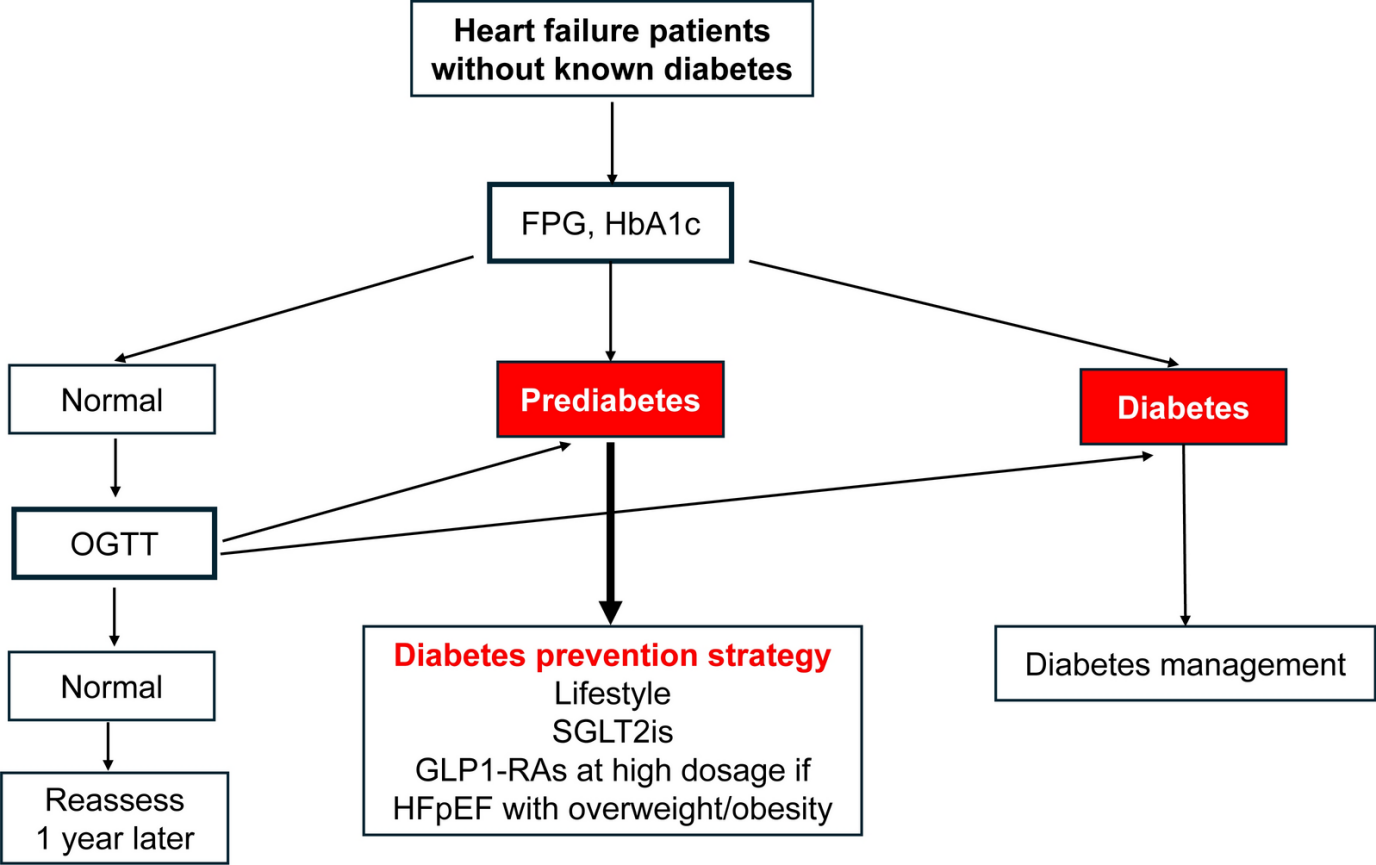
Algorithm for the detection of glucose abnormalities and diabetes prevention in patients with heart failure

The present review highlights the prediabetes status in patients with HF which represents a worthwhile dysglycemic category to intervene at the very early stage of the natural history of diabetes and thus reduce the progression to diabetes and associated complications. Prediabetes is a common condition in HF, associated with a more severe HF and worse CV prognosis. There is an urgent need in HF patients to improve the early detection of unknown glycemic disorders first with FPG and HbA_1c_ measurement and if normal with an OGTT, to treat earlier diabetes when it is so detected and to set a strategy of diabetes prevention in those with prediabetes, as summarized in Fig. 1. Among the available opportunities, SGLT2is have recently shown consistent benefit in the prevention of diabetes in HF patients with prediabetes and a tremendous global health advantage for CV and kidney outcomes. A class effect might not be excluded when comparing the results obtained with dapagliflozin and empagliflozin. This class of drugs, initially developed as oral glucose-lowering drugs to improve glycemic control, might play a consistent role in diabetes prevention. The exact link between the reduction of incident diabetes and CV benefit remains to be clarified. Further research is needed to confirm the benefit of SGLT2is in dedicated trials in broader prediabetic populations. GLP-1 RAs belong to another class of drugs with promising effects at high dosages in obese patients with HF and potentially in diabetes prevention. Both SGLT2is and GLP-1 RAs may offer a new strategy to prevent the development of diabetes and consequently worsening of HF. Last but not least, this review urges physicians, cardiologists and diabetologists to recognize the potential of the prediabetic stage in patients with HF.

## Data Availability

No datasets were generated or analysed during the current study.
